# Human parvovirus B19-associated hematopathy in HIV disease: need for clinicopathological revisit

**DOI:** 10.7555/JBR.32.2017010

**Published:** 2018-01-26

**Authors:** Idris Abdullahi Nasir, Jessy Thomas Medugu, Amos Dangana

**Affiliations:** 1. Department of Medical Laboratory Services, University of Abuja Teaching Hospital, Gwagwalada, FCT Abuja 234, Nigeria; 2. Department of Medical Microbiology and Parasitology, College of Health Sciences, University of Ilorin, Ilorin 1515, Nigeria; 3. Department of Medical Laboratory Science, University of Maiduguri Medical College, Maiduguri, Borno 234, Nigeria.

Persons living with HIV infection occasionally suffer from anemia due to varying causes. These include the use of zidovudine, malnutrition especially vitamin B12 and iron deficiency, opportunistic infections by *Mycobacterium tuberculosis*, *Pneumocystis jiroveci*, and direct hematological effects of HIV infection itself within the marrow microenvironment. Persistent Parvovirus B19 (B19V) infection is a clinically important and treatable etiology of anemia in HIV-infected persons. B19V is known to show singular tropism and lytic infection of erythroid progenitor cells which may consequently result in transient red cell aplasia (TRCA) and chronic anemia.

The transmission of B19V occurs mainly via respiratory droplets, but it can also be spread by contaminated blood, organ transplantation and vertical transmission^[1]^. After respiratory acquisition of B19V, a short replication occurs in the nasopharyngeal lymphoid tissue, followed by a massive viremia with a viral load that can exceed 10^13^ copies per mL^[2]^. Subsequently, B19V is disseminated throughout the body and enters the bone marrow microenvironment producing a generalized erythroblast infection^[2]^. Lymphopenia, neutropenia, and thrombocytopenia that occur during the acute viremic phase are not clinically significant^[3]^. The etiology of thrombocytopenia in productive B19V infection can be partly explained by the presence of viral replication in thrombocytes, without synthesis of structural proteins^[3]^. Patients with various types of immunosuppression may not be able to clear B19V effectively, which can result in persistent low-titer viremia accompanied by B19V related chronic anemia. Persistent B19V infection may result in severe anemia due to the inability to produce neutralizing antibodies on the conformation-dependent neutralizing epitopes, and consequent persistence of the virus replication^[4]^.

Epidemiologically, B19V only infects humans. At 15 years of age, about 50% of children are positive for anti-B19V IgG; this figure can rise to>90% during geriatric ages. About 25% of severe chronic anemia in HIV/AIDS patients has been attributed to B19V infection^[5]^.

Highly active antiretroviral therapy (HAART) has been shown to stimulate reconstitution of humoral and cell mediated immune competence to various opportunistic infections^[6]^. Hence, B19V infection is a diagnosis of exclusion in patients who have started on HAART and developed anemia, then later not responding to empirical management^[6]^. However, the most common sequela of persistent B19V infection is red cell aplasia resulting in chronic or recurrent anemia with reticulocytopenia^[7]^. In immunocompromised individuals such as HIV/AIDS patients with chronic anemia requiring frequent blood transfusions, B19V infection should be suspected and evaluated.

The diagnosis of B19V infection is established through the following criteria: a bone marrow biopsy microscopy with pure red cell aplasia or hypoplasia with giant pronormoblasts and intranuclear inclusions; anti-B19V IgM seropositivity and/or bone marrow positivity for parvovirus B19 DNA, especially when no alternative explanation for anemia can be demonstrated in affected patients^[1]^.

A study by Gyllensten *et al*. detected Parvovirus B19V DNA by a polymerase chain reaction in 69 anemic HIV/AIDS patients. Five out of 69 had detectable B19V DNA and none of 39 non- anemic HIV patients had B19V^[8]^. In another report, Naides *et al*. also examined serial sera from 14 HIV patients with PCR, 9 (64.3%) had detectable viremia at some point by PCR. Four of the 9 had serially B19V positive samples. Since all four with serially positive samples had severe anemia, the author concluded that B19V infection could be the cause of anemia in the patients with HIV/AIDS^[9]^.

The mechanisms that explain the hemopathy of B19V on HIV infected persons have raised considerable concern of B19V associated severe anemia. Knowing whether an anemic HIV patient is co-infected with B19V, prioritizes the need for diagnosis and careful selection of antiretroviral drug in order to prevent exacerbated hematotoxic effects such as severe aplastic anemia.

## References

[1] Heegaard ED, Brown KE. Human parvovirus B19[J]. , 2002, 15(3): 485–505 . 12097253 10.1128/CMR.15.3.485-505.2002 PMC118081

[2] Anderson MJ, Higgins PG, Davis LR, Experimental parvoviral infection in humans[J]. , 1985, 152(2): 257–265 . 2993431 10.1093/infdis/152.2.257

[3] Pallier C, Greco A, Le Junter J, The 3′ untranslated region of the B19 parvovirus capsid protein mRNAs inhibits its own mRNA translation in nonpermissive cells[J]. , 1997, 71(12): 9482–9489 . 9371610 10.1128/jvi.71.12.9482-9489.1997 PMC230254

[4] Young NS, Brown KE. Parvovirus B19[J]. , 2004, 350(6): 586–597 . 14762186 10.1056/NEJMra030840

[5] Fuller A, Moaven L, Spelman D, Parvovirus B19 in HIV infection: a treatable cause of anemia[J]. , 1996, 28(3): 277–280 . 8912362 10.1080/00313029600169154

[6] Gadwalkar SR, Deepa DV, Katageri A, Primary human parvovirus B19 infection in an HIV infected patient on antiretroviral therapy[J]. , 2013, 61(12): 910–912 . 24968551

[7] Hayden TH, Carroll KC, Tang YW, Diagnostic microbiology of the immunocompromised host[M]. [J]. Washington DC: ASM Press, 2009.

[8] Gyllensten K, Sönnerborg A, Jorup-Rönström C, Parvovirus B19 infection in HIV-1 infected patients with anemia[J]. , 1994, 22(5): 356–358 . 7843816 10.1007/BF01715548

[9] Naides SJ, Howard EJ, Swack NS, Parvovirus B19 infection in human immunodeficiency virus type 1-infected persons failing or intolerant to zidovudine therapy[J]. , 1993, 168(1): 101–105 . 8515096 10.1093/infdis/168.1.101

